# Immune correlates of HIV-1 rebound during broadly neutralizing antibody treatment in young children

**DOI:** 10.1172/JCI193912

**Published:** 2026-02-16

**Authors:** Aischa Niesar, Melanie Lancien, Seohyun Hong, Chloe Naasz, Gbolahan Ajibola, Kenneth Maswabi, Maureen Sakoi-Mosetlhi, Oganne Batlang, Sikhulile Moyo, Terence Mohammed, Comfort Maphorisa, Leah Carrere, Isabelle Roseto, Ciputra Adijaya Hartana, Toong Seng Tan, Ce Gao, Elizabeth Parsons, Renee Hua, Molly Pretorius Holme, Shahin Lockman, Kathleen M. Powis, Mary Carrington, Joseph Makhema, Xu G. Yu, Daniel R. Kuritzkes, Roger L. Shapiro, Mathias Lichterfeld

**Affiliations:** 1 Ragon Institute of MGH, MIT and Harvard, Cambridge, Massachusetts, USA.; 2 Infectious Disease Division, Brigham and Women’s Hospital, Boston, Massachusetts, USA.; 3 Botswana Harvard Health Partnership, Gaborone, Botswana.; 4 Department of Immunology and Infectious Diseases. Harvard T.H. Chan School of Public Health, Boston, Massachusetts, USA.; 5 School of Allied Health Professions, Faculty of Health Sciences, University of Botswana, Gaborone, Botswana.; 6 School of Health Systems and Public Health, University of Pretoria, Pretoria, South Africa.; 7 Division of Medical Virology, Faculty of Medicine and Health Sciences, Stellenbosch University, Cape Town, South Africa.; 8 Basic Science Program, Frederick National Laboratory for Cancer Research, and; 9 Laboratory of Integrative Cancer Immunology, Center for Cancer Research, National Cancer Institute, Bethesda, Maryland, USA.

**Keywords:** AIDS/HIV, Immunology, Immunotherapy, Innate immunity, NK cells

## Abstract

Broadly neutralizing antibodies (bnAbs) are evaluated as possible alternatives to standard antiretroviral treatment (ART) for maintaining control of HIV-1 replication and may enhance immune responses to reduce or control the viral reservoir. However, the immunological and virological effects of bnAbs in infants and children are unknown. We conducted a detailed analysis of proviral reservoir dynamics and antiviral immune responses in a unique group of young children from Botswana who started ART at birth and then stopped standard ART while receiving the bnAbs 10-1074 and VRC01-LS in a subsequent clinical trial. No quantitative changes in frequencies of proviral sequences were observed during bnAb treatment, but selection of genome-intact proviruses in transcriptionally repressive heterochromatin regions occurred in some study participants. Faster viral rebound following standard ART cessation was linked to elevated proportions of KIR2DL1-positive NK cells. In contrast, delayed viral rebound and more limited viral reservoir size were associated with elevated proportions of NKG2A-positive NK cells and with the HLA-B-21M signal peptide polymorphism. HIV-specific T cell responses were low in all study participants and unrelated to viral reservoir sizes or clinical outcomes following ART interruption. These results suggest that, in young children, specific NK cell subsets and KIR-HLA interactions might be linked to HIV-1 rebound kinetics after substitution of standard ART with bnAbs.

## Abstract 2

In a clinical trial of young children with HIV receiving broadly neutralizing antibodies, natural killer cell profiles were associated with viral rebound and reservoir size, suggesting a possible role in viral immune control

## Introduction

Pediatric HIV-1 infection can occur by vertical transmission in utero, during childbirth, or through breastfeeding and currently affects more than 1.4 million children worldwide (1). While antiretroviral agents for pediatric use are now readily available in most parts of the world, they are typically not formulated as single-pill regimens and can be associated with side effects and marked adherence challenges (2, 3). As a result, fewer infants and children achieve viral suppression than adults and the mortality rate for pediatric HIV-1 infection remains high, even when antiretroviral therapy (ART) is initiated early in life (4–8).

Broadly neutralizing antibodies (bnAbs) are an emerging treatment modality for people living with HIV-1 and can effectively suppress HIV-1 plasma viremia in adults with susceptible viral strains (9, 10). Potential advantages of bnAbs over standard small-molecule ART include less frequent dosing, reduced long-term toxicities, and the putative ability of bnAbs to engage host immune responses against HIV-1 reservoir cells that persist despite antiretroviral therapy (11). In the pediatric setting, bnAbs may represent an attractive long-acting treatment modality with limited side effects that could facilitate treatment adherence and improve clinical outcomes. However, very few studies have evaluated the possible clinical benefits of bnAb therapy in infants and young children. Moreover, immune effects of bnAbs in infants and children and their presumed abilities to amplify antiviral host immune responses are currently unknown.

Recent studies have identified several adults who maintained drug-free control of HIV-1 infection after treatment with bnAbs, possibly due to the ability of bnAbs to increase antiviral host immune function or restrict viral rebound from persisting viral reservoirs (12–15). Reductions of viral reservoir cells during bnAb therapy have also been reported (15). Prior animal experimentation studies and human clinical trials in adults suggest that bnAb treatment may be associated with an improved HIV-1–specific T cell response, possibly through a bnAb-mediated enhancement of antigen-presenting and costimulatory properties of myeloid dendritic cells (16–18). Some studies in adults suggest that this vaccinal effect can translate into a clinically significant extension of drug-free HIV-1 control following treatment interruptions (19). However, HIV-1–specific T cells in infants and children starting ART early after pre- or perinatal infection are typically weak, likely due to the immaturity of the adaptive immune system in early life (20–23). Whether and how innate immune effector cell responses can modulate the effects of bnAbs in infants and young children has not been studied.

The Tatelo study (24) was a phase I/II single-arm, multisite clinical trial to evaluate the combined use of 2 bnAbs, VRC01-LS and 10–1074, in young children from Botswana who acquired HIV-1 by in utero vertical transmission and started standard ART shortly after birth (20, 25). During the Tatelo study, both bnAbs were administered monthly for at least 2 months in conjunction with standard ART; subsequently, small-molecule ART was stopped, and bnAb treatment was continued for a maximal duration of 6 months before switching back to standard ART. If a single plasma viral load of greater than 400 copies/mL (identified in 2 repetitive samples) occurred during bnAb-only treatment, standard ART was reinstituted immediately. Of the 25 children enrolled, 11 completed the entire 6 months of bnAb-only treatment, while 14 experienced viral rebound earlier (24). Previous work identified several nonimmunologic factors that were associated with viral control in the Tatelo cohort, including viral reservoir size at birth, number of blips or viral rebounds in early life, and viral susceptibility to administered bnAbs, especially 10-1074 (24). In this study, we describe a detailed analysis of changes in viral reservoir cells and immune responses during bnAb therapy in the Tatelo study that were experimentally obtained using the small blood volumes that can be safely obtained from young children.

## Results

### Longitudinal viral reservoir dynamics.

To investigate how bnAb therapy influenced viral reservoir cell evolution in the Tatelo study, we focused on the analysis of 25 study participants who completed the entire study protocol (Supplemental Figure 1; supplemental material available online with this article; https://doi.org/10.1172/JCI193912DS1). The clinical trial started with an at least 8-week period of combined bnAb and ART treatment (step 1), followed by up to 6 months of bnAb-only treatment (step 2), and a follow-up period of up to 3 years after reinitiation of ART (step 3 and follow-up). Clinical, demographical, and immunogenetic data of the study participants included in the analysis are summarized in Supplemental Tables 1–3. During Step 2, 11 participants (“controllers”) maintained viral loads below 400 copies/mL for the full 6-month period, while 14 participants (“rebounders”) experienced virological rebound, leading to earlier ART reinitiation and discontinuation of bnAb treatment. The median time to ART reinitiation in rebounders was 4 weeks but varied among individual study participants (range 1–20 weeks). For detailed virological studies, PBMC samples were available at baseline prior to bnAb-only treatment (step 1, during combined ART and bnAb treatment), from immediately prior or at the time of ART reinitiation (end of step 2; controller: 24 weeks of dual bnAb treatment, rebounder: rebound timepoint) and at multiple follow-up timepoints after ART resumption (throughout and after step 3). Using these samples, we performed near full-length individual proviral sequencing (FLIP-seq) to quantify viral DNA sequences, to distinguish intact and defective proviruses, and to identify clonal sequences based on proviral genome sequence identity (26). These data were complemented by proviral sequences obtained by an identical experimental protocol from blood samples collected from the study participants immediately after birth and over the first 2 years of life, as described in our previous work (20).

**Figure 1:**
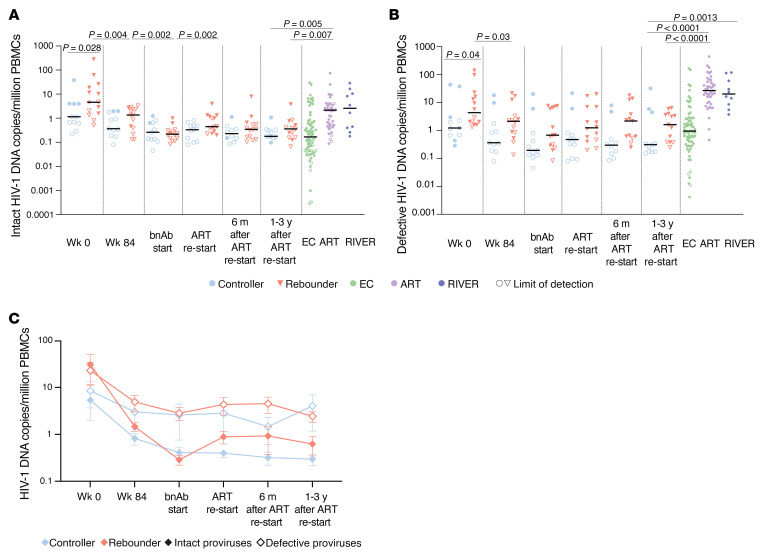
Viral reservoir dynamics in participants of the Tatelo study. (**A** and **B**) Frequency of intact (**A**) and defective (**B**) proviruses in controllers and rebounders from the Tatelo study, measured at indicated timepoints (wk0, birth; wk84, week 84 after birth). Data from elite controllers (EC), long-term ART-treated adults who initiated treatment during chronic infection, and adults who started ART during acute infection in the RIVER cohort (27) are presented for comparative purposes. Limit of defection (LOD) was calculated as 0.5 copies per maximum number of cells tested without target identification. Data were obtained by near full-length proviral sequencing (FLIP-Seq) or by matched integration site and proviral sequencing (MIP-Seq). (**C**) Decay kinetics of intact and defective proviral HIV-1 sequences before, during, and after the Tatelo study. Mean and SEM are shown. (**A** and **B**) Significance was tested using 2-tailed Mann-Whitney-U or Wilcoxon matched-pairs signed-rank tests; nominal *P* values are indicated. (**A** and **B**) Medians are indicated by horizontal lines.

**Figure 2:**
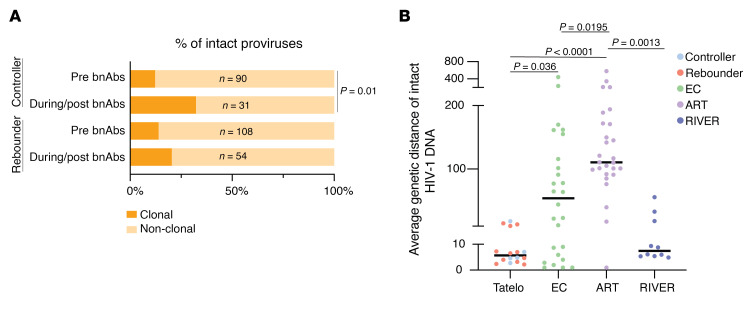
Clonality and genetic distance of intact proviruses in the Tatelo cohort. (**A**) Bar graphs reflecting proportions of intact proviruses detected once (nonclonal) or multiple times (clonal) in the controller and rebounder group before bnAb treatment and after Tatelo entry. (**B**) Average genetic distance of intact proviruses from the Tatelo cohort (*n* = 16), ART-treated adults (*n* = 27), Elite controllers (*n* = 26) and participants of the RIVER study (*n* = 10) determined by pairwise comparisons between all intact sequences within each study person. Data from all Tatelo participants with at least 2 different intact proviruses were included. χ^2^ test was used in **A**. Significance was tested using 2-tailed Mann-Whitney-U or Wilcoxon matched-pairs signed-rank tests; nominal *P* values are indicated (**B**). Medians are indicated by horizontal lines (**B**).

**Figure 3:**
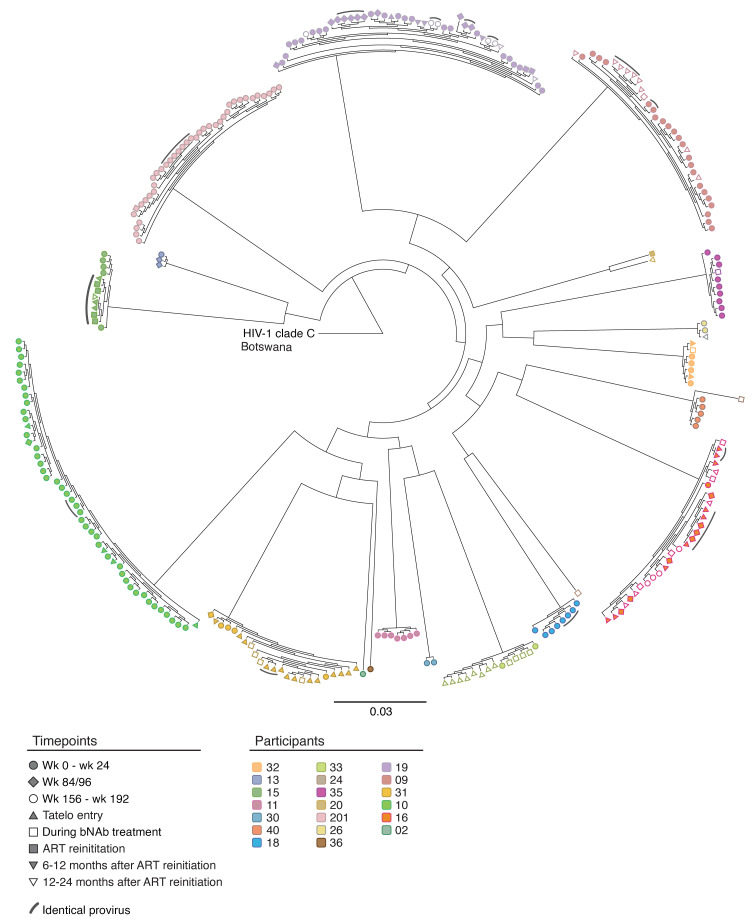
Circular maximum-likelihood phylogenetic tree of intact proviral sequences from participants in the Tatelo cohort. A clade C HIV-1 sequence from Botswana was used as reference for clade C. Clonal sequences, defined by complete sequence identity, are highlighted by arches. Timepoints of sample collection are indicated by symbols.

In total, we analyzed a median of approximately 1.55 million (range: 8.10 × 10^5^–2.89 × 10^6^) PBMCs per participant per timepoint and identified 85 intact and 1,886 defective proviruses across all Tatelo timepoints in both groups combined. Total numbers of analyzed PBMCs and obtained sequences are summarized in Supplemental Table 5. In 3 of the 25 study participants, we failed to detect any type of intact or defective proviruses, likely due to limited PBMC quantities that could be safely obtained from infants and young children, and because viral reservoirs in our study participants with ART initiation at birth were generally low and markedly smaller than viral reservoirs in adults. Despite these limitations, we observed that controllers had significantly lower levels of intact and defective proviruses at birth compared with rebounders (Figure 1, A and B, and Supplemental Figure 2). No quantitative differences were noted between the 2 study groups at week 84 after ART initiation, or at the beginning of bnAb-only treatment in the Tatelo study. However, at ART reinitiation after the bnAb-only treatment, there was a significant increase of intact proviruses in the rebounder group compared with the start of bnAb therapy, as expected (Figure 1, A and B). Notably, intact proviruses decreased in most rebounders 6 months after ART reinitiation, suggesting that newly infected cells after viral rebound were unstable and, to some extent, subject to subsequent immune clearance. At the latest follow-up timepoint at a median of 26 months after ART reinitiation (range 17–36 months), levels of intact and defective proviruses in Tatelo study participants were substantially lower compared with a reference cohort of adults who initiated treatment during acute or chronic infection (Supplemental Table 4), emphasizing the small viral reservoir size in infants who started treatment early in life. Frequencies of intact proviruses from the Tatelo cohort at the last analysis timepoint were not different from a reference adult cohort of elite controllers (EC) but were lower compared with long-term ART-treated adults and to adults on short-term ART started in acute infection from the RIVER cohort (27) (Figure 1, A and B, and Supplemental Table 4). Consistent with prior results (28–30), we noted that, in both rebounders and controllers, the frequencies of intact proviruses declined more rapidly during the initial year after ART initiation compared with defective proviruses; however, there were no kinetic differences in the decay rates of intact proviruses between the 2 study groups (Figure 1C), and we detected no experimental evidence supporting a decrease of viral reservoir cells during bnAb therapy. The proportions of clonal genome-intact sequences (defined as sequences detected at least 2 times at a given timepoint) within the total pool of intact sequences were not different prior to bnAb treatment between controllers and rebounders. While both groups showed a proportional increase in clonal intact sequences over time, this was more pronounced and reached statistical significance only in the controller group (Figure 2A). Generally, proviral sequence diversity in the Tatelo cohort was very low and did not differ between both groups, as determined by calculating pair-wise genetic distances between individual intact proviruses in each study participants (Figure 2B and Figure 3). In summary, our findings demonstrate extremely low levels of intact proviruses at most timepoints for Tatelo participants, which limited our ability to evaluate the impact of bnAb treatment on viral reservoirs in this cohort of infants and young children.

### Chromosomal location of HIV-1 proviral sequences.

**Figure 4:**
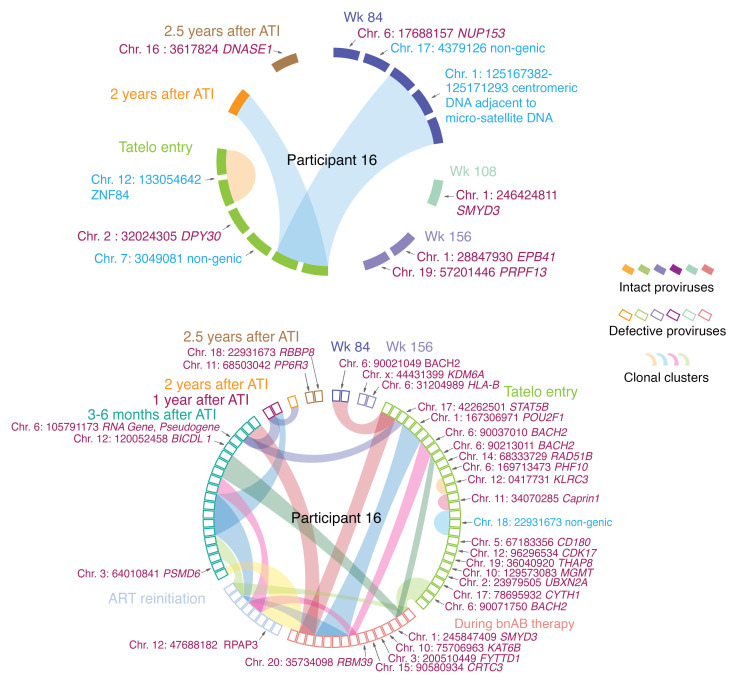
Proviral integration site analysis in Tatelo study participant 16. CIRCOS plots reflect the chromosomal locations of intact (filled symbols) or defective proviruses (outline symbols) at the indicated time points in study participant 16. Each symbol reflects 1 intact or defective provirus. Clonal sequences, defined by identical integration sites and/or complete sequence identity, are highlighted. Only sequences for which integration sites were available are shown. Purple font color indicates chromosomal locations in genes; blue font color indicates chromosomal integration sites in non-genic/heterochromatin locations.

For a deeper analysis of qualitative changes in the viral reservoir cell pool, we employed matched integration site and proviral sequencing (MIP-seq), a method allowing for identifying the chromosomal integration sites of defined proviral sequences. This experimental approach has previously been used to unravel longitudinal selections of intact proviruses integrated in heterochromatin regions during long-term ART in adults (31). We identified a total of 121 integration sites from 3 controllers from whom sufficient cell quantities were available, permitting a deeper analysis. All integration sites are listed in Supplemental Table 6. In controller participant 16 (Figure 4), we observed clonal expansion of cells harboring an intact provirus integrated into centromeric satellite DNA of chromosome 1, a chromatin region that displays repressive chromatin features (32). This clone was detected at week 84 after birth, at the beginning of the Tatelo study, and 2 years after the Tatelo study. In addition, a clone of cells harboring intact proviruses integrated into the ZNF gene 84, a genomic location also associated with repressive chromatin characteristics (33), was observed at the time of Tatelo entry. For comparative purposes, we also analyzed integration sites of a larger number of defective proviruses in study participant 16 (Figure 4). Notably, none of these defective proviruses were integrated in centromeric satellite DNA or in ZNF genes; instead, defective proviruses in this individual were mostly located in highly expressed genes that are preferred sites for viral integration. Four distinct clones of defective proviruses in study participant 16 were integrated in the *BACH2* gene. This is in line with prior findings indicating that integration into the *BACH2* gene locus can support viral persistence, presumably due to retroviral insertional mutagenesis leading to a *BACH2*-mediated increase in proliferative turnover of cells harboring integrated proviral DNA at this genomic location (34, 35).

**Figure 5:**
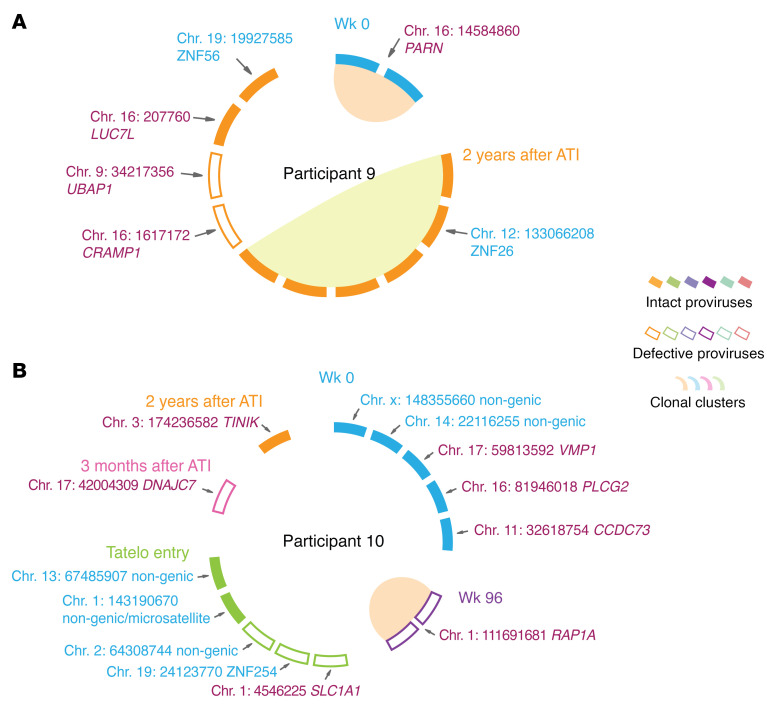
Proviral integration site analysis in Tatelo study participants 9 and 10. CIRCOS plots reflect the chromosomal locations of intact (filled symbols) or defective proviruses (outline symbols) at indicated time points in study participants 9 (**A**) and 10 (**B**). Each symbol reflects one intact or defective provirus. Clonal sequences, defined by identical integration sites and/or complete sequence identity, are highlighted. Only sequences for which integration sites were available are shown. Purple font color indicates chromosomal locations in genes; blue font color indicates chromosomal integration sites in non-genic/heterochromatin locations.

In 2 additional controller study participants, we also noted persistence of intact proviruses in ZNF genes: in study participant 9, we observed one large clone of intact proviruses integrated in ZNF gene 26 and an additional proviral sequence in ZNF gene 56, 2 years after the Tatelo study (Figure 5A). In study participant 10, intact proviruses in nongenic regions were detected after birth and at the time of Tatelo entry (Figure 5B). Of note, no clonal expansion of intact proviruses integrated in genes was detected after the first 12 weeks of life in these 3 controller study participants. Defective proviruses integrated in nongenic regions or in ZNF genes were not detected in study participant 9, and in only 2 defective proviruses from study participant 10. Although being limited by the relatively small numbers of proviruses that could be precisely mapped to chromosomal locations in the small amount of PBMCs available for analysis from infants, these results suggest preferential persistence of intact proviruses in nongenic regions and ZNF genes. This is likely because viral integration in such repressive heterochromatin regions effectively suppresses HIV-1 gene transcription, permitting avoidance of host immune recognition. Therefore, the longitudinal selection of intact proviruses in repressive chromatin can be interpreted as a sign of host immune pressure against viral reservoir cells in infants.

### KIR2DL1-expressing NK cells distinguish controllers from rebounders.

To better explore immune mechanisms that may be involved in targeting HIV-1 reservoir cells and modulating HIV-1 rebound kinetics during the Tatelo study, we initially focused on NK cells, which play a critical role in immune defense against viral infections, particularly in the infant immune system that typically lacks fully developed adaptive B and T cell responses (36). To investigate NK cell responses and their possible association with viral reservoir and viral rebound during bnAb therapy in infants, we conducted detailed multiparametric flow cytometry experiments in PBMC samples collected immediately prior to bnAb-only treatment and at the time of ART reinitiation. In an initial analysis, we identified 5 NK cell subsets according to CD16 and CD56 surface expression (Supplemental Figure 3A and Supplemental Figure 4). We observed a slight nonsignificant expansion of CD56^dim^ CD16^dim^ NK cells in the controller group when compared with the rebounder group (Supplemental Figure 3B). This specific NK cell subset is typically associated with higher cytotoxic activity (37). Other NK cell subsets defined by CD16 and CD56 expression patterns, such as CD56^bri^ CD16^low^, CD56^–^ CD16^dim^, CD56^pos^ CD16^–^, and CD56^–^ CD16^bri^ NK cells, did not quantitatively differ between the 2 groups of study participants. Of note, no marked differences in NK cell subsets were observed between the beginning and the end of bnAb-only therapy within each of the study groups (Supplemental Figure 3B). However, the proportions of CD56^dim^ CD16^dim^ NK cells slightly decreased after discontinuation of standard ART.

**Figure 6:**
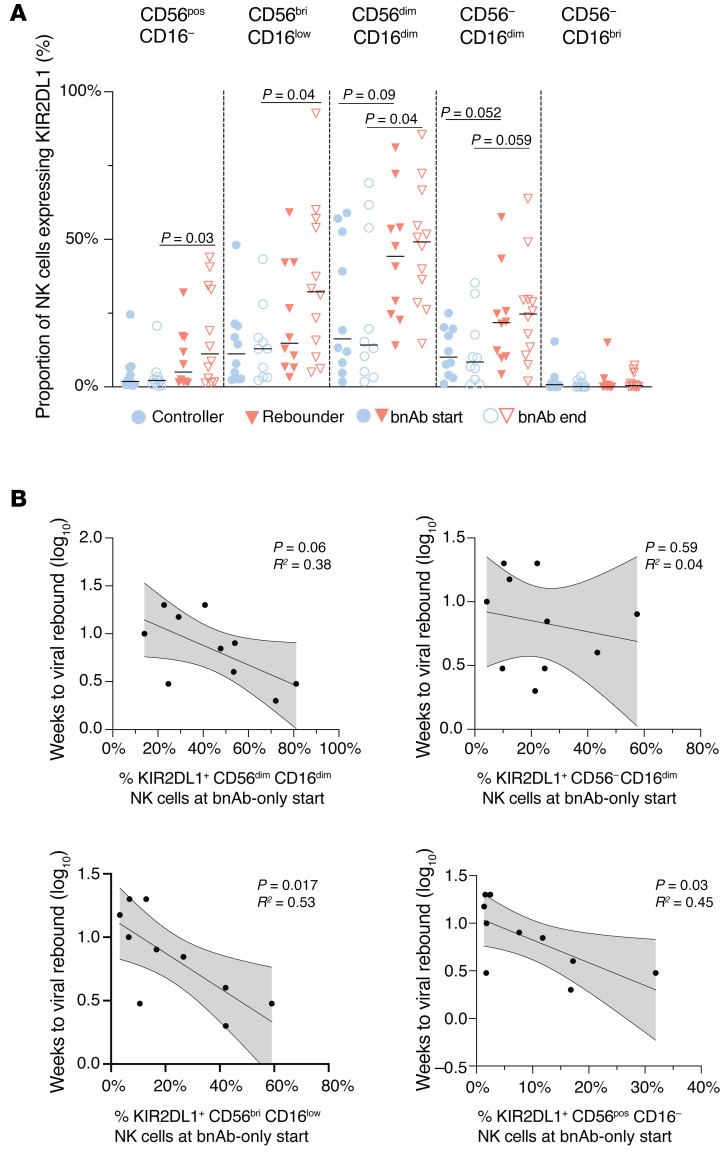
Expression of KIR2DL1 on NK cells in the controller and rebounder group. (**A**) Relative expression of KIR2DL1 in 5 distinct NK cell subsets (defined by CD16 and CD56 expression) in the controller and rebounder group at Step 2 start (bnAb start) and Step 2 end (bnAb end). Medians are indicated by horizontal lines. (**B**) Association between indicated NK cell subsets expressing KIR2DL1 (measured at the beginning of step 2) and weeks to viral rebound (rebounder group). Linear regression coefficients with nominal *P* values are shown. Mann-Whitney-U test was used in **A**. Nominal *P* values are indicated.

We subsequently focused on analyzing NK cells expressing Killer Immunoglobulin-Like Receptors (KIRs), markers that play an important role in NK cell education and licensing (38). While KIR3DL1, KIR2DL5, and KIR2DL2 showed no differences in NK cell surface expression between our 2 groups of participants, we observed that the frequency of NK cells expressing KIR2DL1, the major inhibitory NK cell receptor for HLA-C2 allotypes, was significantly elevated in the rebounder group at ATI conclusion (end of Step 2) in the following NK cell subsets: CD56^pos^ CD16^–^, CD56^bri^ CD16^low^, and CD56^dim^ CD16^dim^ (Figure 6A). We also noted that the proportion of KIR2DL1-expressing NK cells, determined immediately prior to stopping standard ART (step 1), tended to negatively correlate with viral rebound kinetics in the CD56^dim^ CD16^dim^ NK cells (*P* = 0.06), in the CD56^bright^ CD16^low^ NK cell subset (*P* = 0.017), and in the CD56^pos^ CD16^–^ NK cell subset (*P* = 0.03) (Figure 6B). These data show that higher levels of KIR2DL1 expression on NK cells were associated with accelerated viral rebound during bnAb therapy.

**Figure 7:**
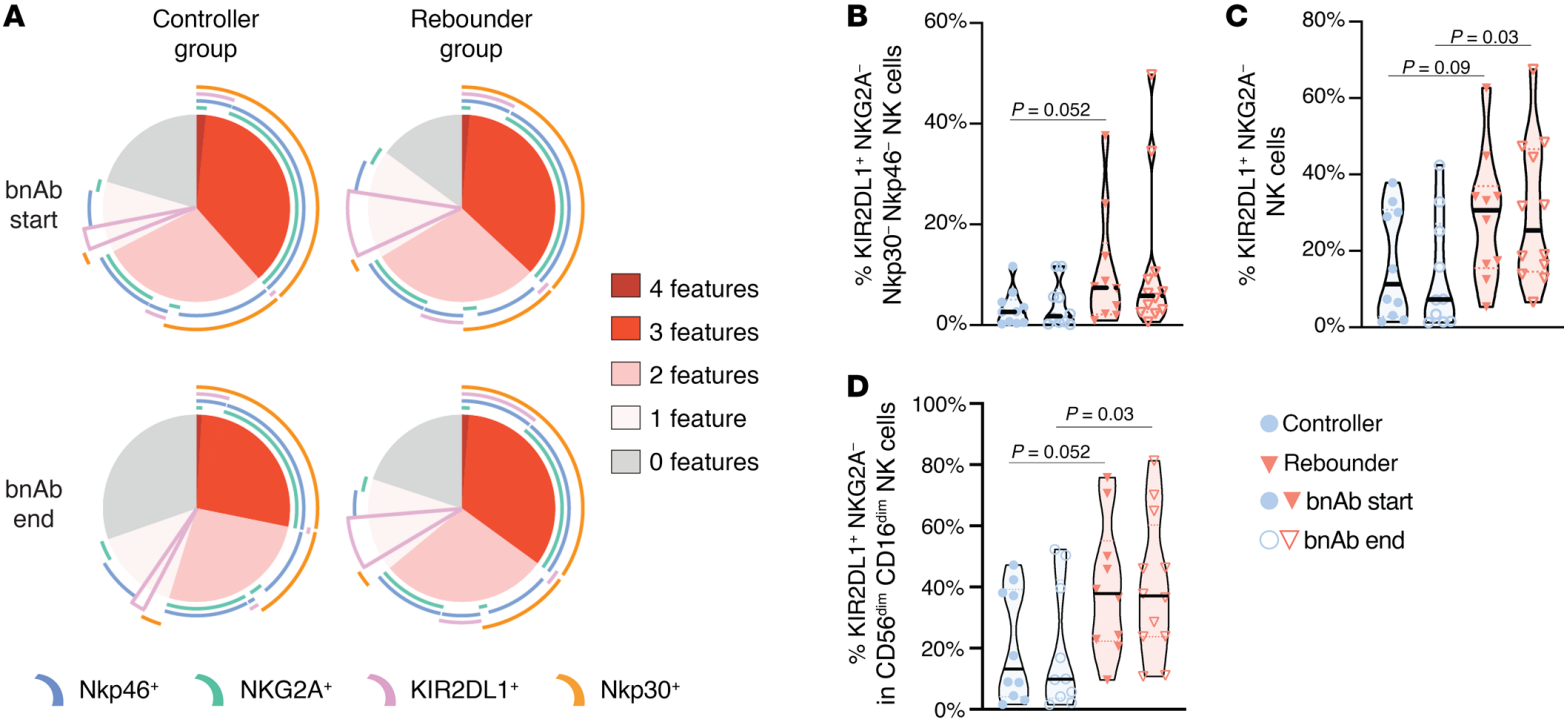
Combinatorial surface expression of KIR2DL1, Nkp30, NKG2A, and Nkp46 on NK cells in the controller and rebounder group. (**A**) SPICE diagrams reflect proportions of NK cells expressing KIR2DL1 in the presence or absence of Nkp46, NKG2A, and Nkp30 coexpression (determined by Boolean combination gating) in the controller and the rebounder group. Pie chart color represents number of expressed receptors, whereas each receptor is represented by an arc. (**B**–**D**) Frequency of KIR2DL1^+^ NK cells expressing the indicated phenotypic profile in the controller and rebounder group at Step 2 start and Step 2 end. Mann-Whitney-U test was used in **B**–**D**. Nominal *P* values are indicated.

To further evaluate connections between KIR2DL1-expressing NK cells and viral rebound kinetics, we assessed KIR2DL1 surface expression in the context of additional immunomodulatory NK cell markers. These studies demonstrated that proportions of KIR2DL1-expressing NK cells lacking the expression of NKp30/NKp46 and NKG2A were higher in rebounders than in controllers (Figure 7). This was also true when KIR2DL1^+^ NKG2A^–^ NK cells were evaluated independently of NKp30/NKp46 expression and was most notable in the dominant CD56^dim^ CD16^dim^ NK cell subset.

### NKG2A^+^ NK cell expression patterns during bnAb treatment.

**Figure 8:**
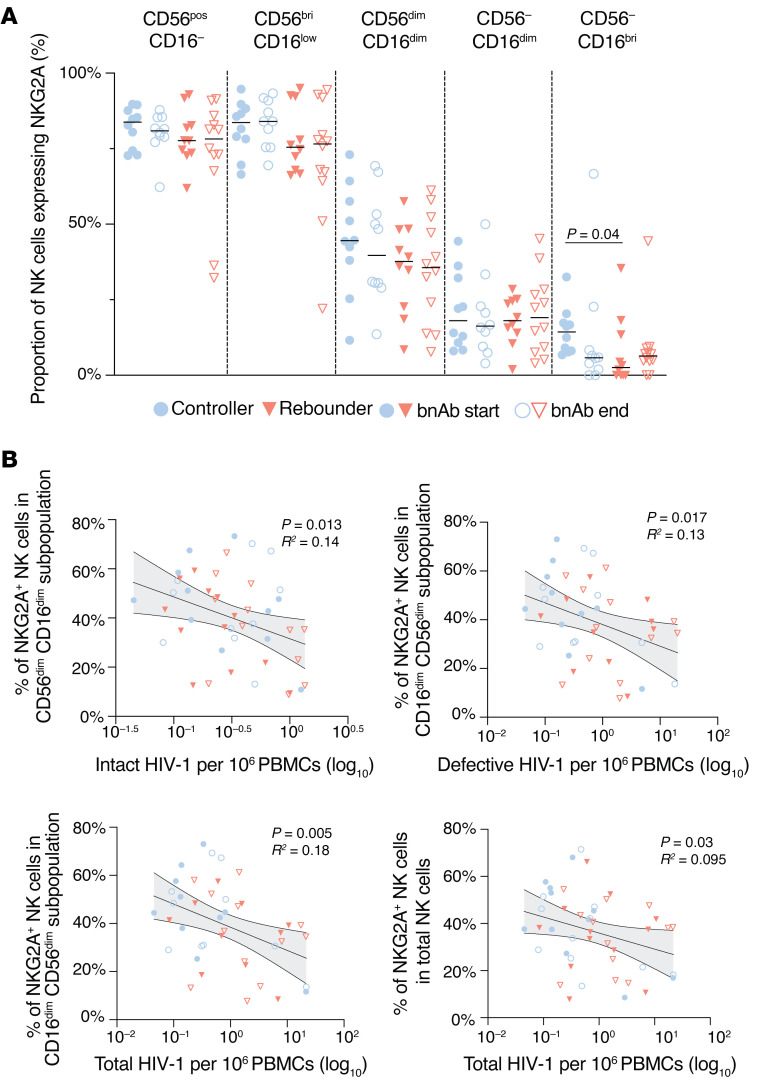
Frequencies of NKG2A-expressing NK cells in Tatelo study participants. (**A**) Proportions of NKG2A^+^ cells in 5 distinct NK cell subsets (defined by CD16 and CD56 expression) in the controller and rebounder group at the start and the end of Step 2. Medians are indicated by horizontal lines. (**B**) Association between indicated NKG2A-expressing NK cell subsets and frequency of intact, defective, or total proviruses (data from both study groups at Step 2 start and Step 2 end, LOD values were included).

**Figure 9:**
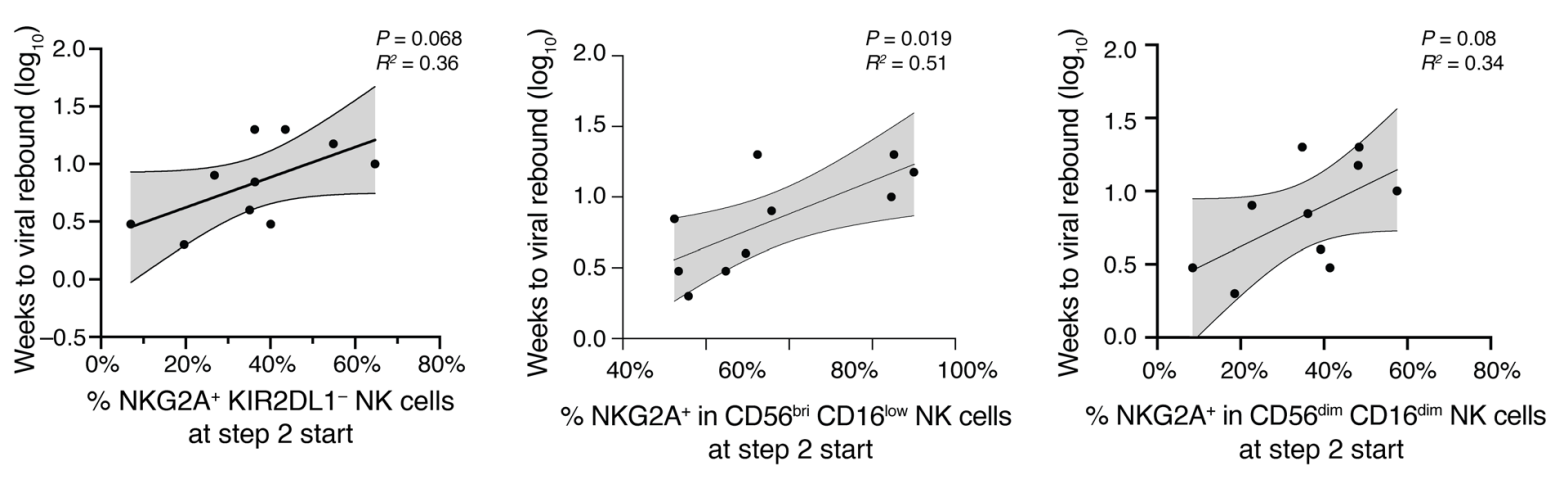
Correlation of NKG2A^+^ KIR2DL1^–^ NK cells and viral rebound kinetics. Data show the association of NKG2A^+^ KIR2DL1^–^ NK cells (measured at the beginning of step 2) and weeks to viral rebound (rebounder group). Linear regression coefficients with nominal *P* values are shown.

We subsequently focused on NK cells expressing NKG2A, an inhibitory marker that influences NK cell education and function through interactions with HLA-E, and has been associated with superior cytotoxic activities against HIV-1–infected cells in some studies (39–41). We found no marked differences in NKG2A expression between NK cells from controllers and rebounders (Figure 8A); this was also noted for frequencies of NK cells expressing the activating receptor NKG2C, which also recognizes HLA-E (Supplemental Figure 3C). Nevertheless, the proportions of NKG2A-expressing cells within the dominant CD56^dim^ CD16^dim^ NK cell subset were inversely correlated with the frequencies of intact and defective proviral copies (Figure 8B); moreover, proportions of NKG2A^+^ cells within the total NK cell compartment were also inversely associated with total proviral copy frequencies. In addition, fractions of NKG2A^+^ NK cells, measured prior to ART interruption, tended to correlate with the time to viral rebound following ART discontinuation; in particular, this was true for KIR2DL1^–^ NKG2A^+^ NK cells (*P* = 0.068), for NKG2A^+^ CD56^dim^ CD16^dim^ NK cells (*P* = 0.08), and for NKG2A^+^ CD56^bri^ CD16^low^ NK cells (*P* = 0.019) (Figure 9). Together, these results suggest that higher frequencies of NKG2A-expressing NK cells are linked to delayed viral rebound kinetics during bnAb-only therapy. To complete the characterization of the innate immune cells in our study groups, we analyzed the frequencies of monocytes and their subtypes (classical, intermediate, nonclassical) as well as dendritic cells (mDC, pDC, DC1, DC2-3, DC4). No statistically significant differences were observed between the controller and rebounder groups at the start of bnAb-only treatment or at ART reinitiation (Supplemental Figure 4 and Supplemental Figure 6, D and E), nor were there any associations between these innate immune cell subsets and viral rebound kinetics or viral reservoir sizes.

### Immunogenetic associations.

**Figure 10:**
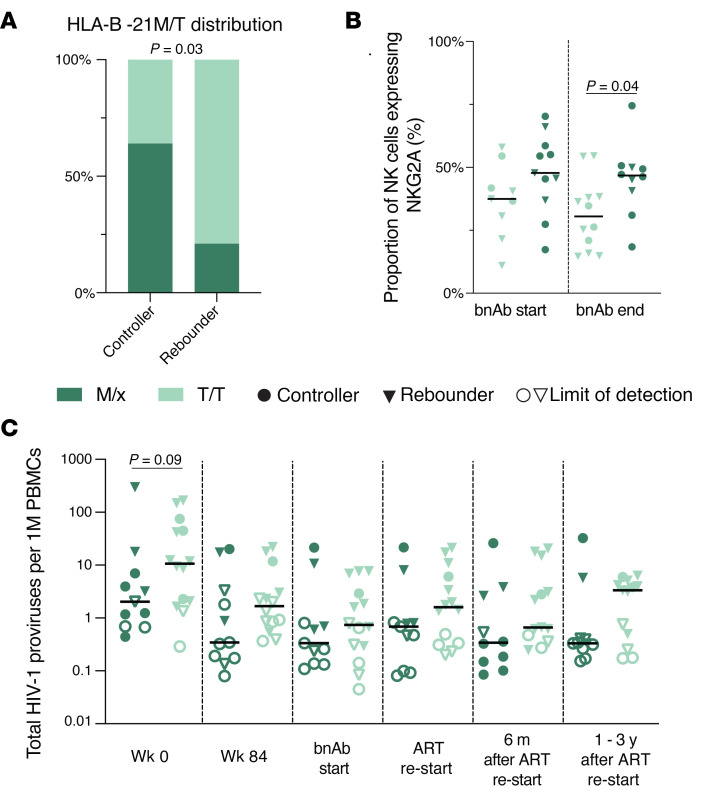
Distribution of the HLA-B21 M/T polymorphism in the Tatelo cohort. (**A**) Proportions of carriers with the M/x or T/T genotype of the HLA-B21 M/T signal peptide polymorphism within rebounders and controllers. (**B**) Frequencies of NKG2A-expressing NK cells in carriers of the HLA-B21 T/T genotype or carriers of at least one -B21M allele (M/x). (**C**) Total HIV-1 proviruses in carriers of the T/T genotypes or in carriers of at least one -B21M allele. χ^2^ test was used in **A**. Mann-Whitney-U test in **B** and **C**. Nominal *P* values are reported. (**B** and **C**) Medians are indicated by horizontal lines.

**Figure 11:**
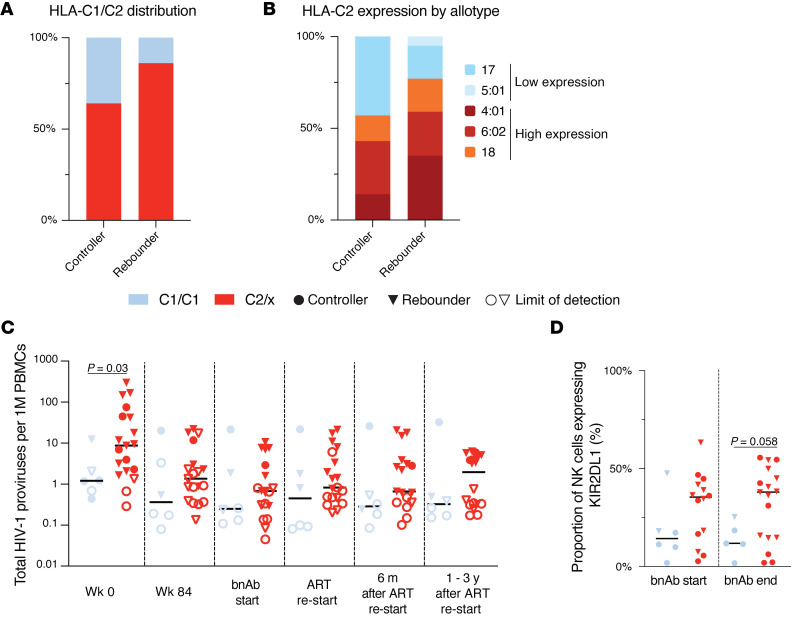
Distribution of HLA-C1/C2 alleles in the Tatelo cohort. (**A**) Proportions of carriers of the HLA-C1/C1 or the HLA-C2/x genotype in the controller and rebounder group. (**B**) Frequencies of indicated HLA-C2 alleles with high or low surface expression in the controller and rebounder group. (**C**) Total HIV-1 proviruses within participants with HLA-C1/C1 alleles or carriers of at least one HLA-C2 allele (C2/x). (**D**) Frequencies of KIR2DL1-expressing NK cells within participants with HLA-C1/C1 alleles or carriers of at least one HLA-C2 allele. Mann-Whitney-U test in **C** and **D**. Nominal *P* values are reported. (**C** and **D**) Medians are indicated by horizontal lines.

To further explore the possible impact of NK cells on viral reservoirs and viral rebound kinetics, we analyzed immunogenetic characteristics known to influence NK cell education and function in our study participants. We observed marked differences in allele frequencies for the HLA-B21M/T signal peptide polymorphism between rebounders and controllers, with T/T genotypes being significantly more prevalent in rebounders, while controllers were more frequently carriers of at least one -B21M allele (*P* = 0.03), (Figure 10A and Supplemental Table 2). The presence of at least one -B21M allele can stabilize HLA-E surface expression and has been reported to promote NKG2A-dependent education of CD56^dim^ NK cells (42). However, compared with alternative signal peptides, the -B21M peptide can reduce the binding affinity of the HLA-E complex to NKG2A, resulting in a lower activation threshold for NKG2A-expressing NK cells (43). We noted that carriers of at least 1 -B21M allele had slightly higher proportions of NKG2A-expressing NK cells and lower levels of HIV-1 proviral copies before, during, and after bnAb therapy (Figure 10, B and C). However, these differences failed to reach statistical significance in our small study cohort. Additional immunogenetic studies demonstrated that carriers of HLA-C2 alleles, encoding for the major ligands of KIR2DL1, were slightly but not significantly more frequent among rebounders compared with controllers (Figure 11A and Supplemental Table 2). In particular, we noted that, among all carriers of HLA-C2 alleles, HLA-C2 allotypes with high surface expression, as experimentally assessed in a prior study (44), were nominally enriched in rebounders, while HLA-C2 allotypes with lower cell surface expression were more frequent in controllers (Figure 11B). Children with at least 1 HLA-C2 allele tended to have higher levels of KIR2DL1-expressing NK cells and displayed an increase in levels of proviral HIV-1 DNA copies at birth, associated with a trend for higher HIV-1 DNA levels at subsequent timepoints (Figure 11, C and D). Genes encoding for the activating receptor KIR2DS1 were only present in a minority of our study persons (5 of 25, 20%) and were unrelated to proviral reservoir size or time to viral rebound; the same was true for the presence or absence of KIR-A or KIR-B haplotypes (Supplemental Table 3 and Supplemental Figure 3D). Together, these immunogenetic data raise the possibility that the HLA-B-21M/T polymorphism and the presence or absence of HLA-C1/2 alleles may have influenced the phenotypic profile of NK cells, the proviral reservoir, and the propensity for viral rebound in Tatelo study participants.

### HIV-1–specific T cells during bnAb treatment.

**Figure 12:**
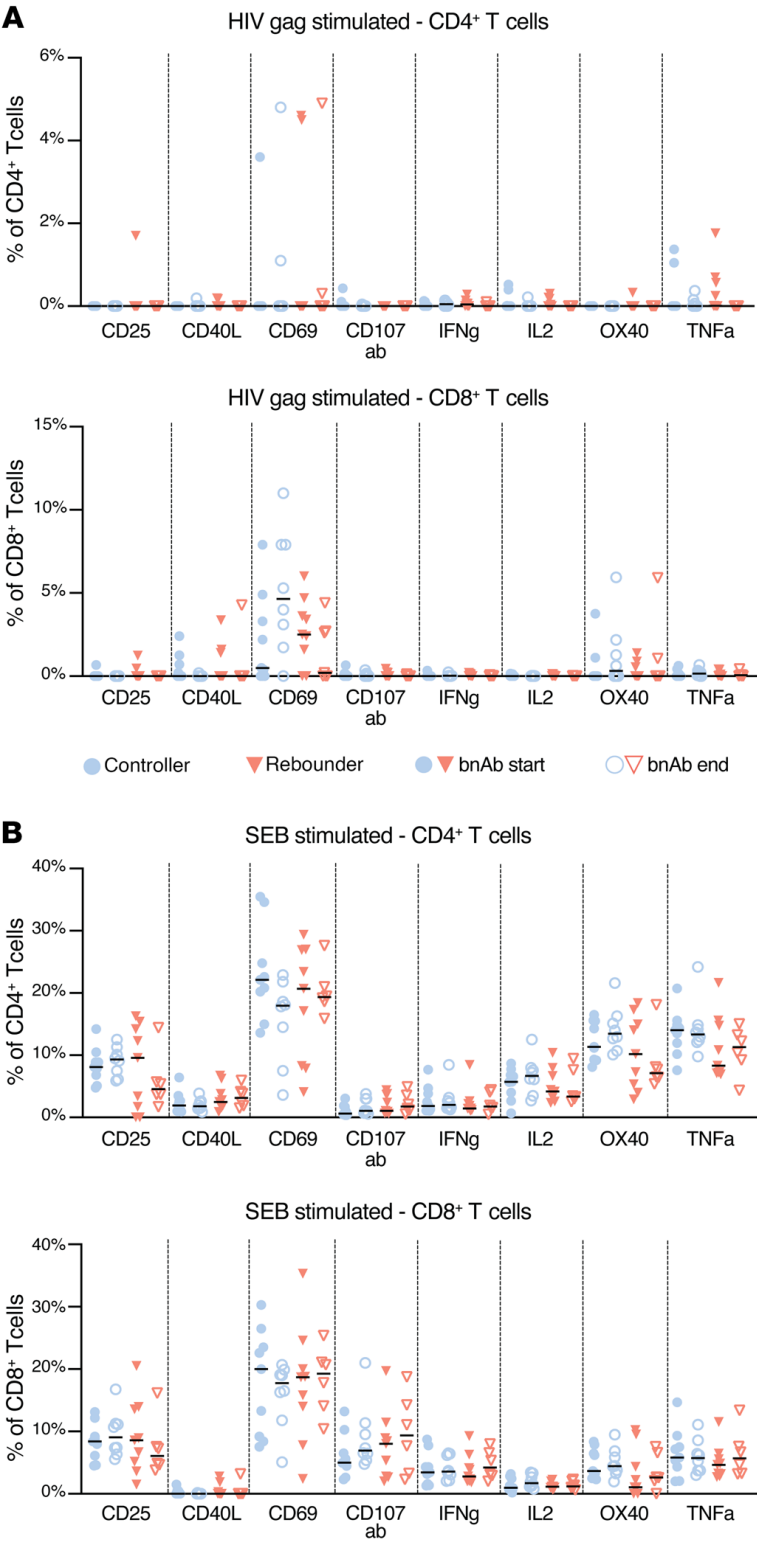
HIV-1–specific CD4^+^ and CD8^+^ T cell responses in the controller and rebounder group. (**A** and **B**) Frequencies of CD4^+^ and CD8^+^ T cells expressing selected activation markers and cytokines after stimulation with HIV-1 gag or SEB. Medians are shown.

**Figure 13:**
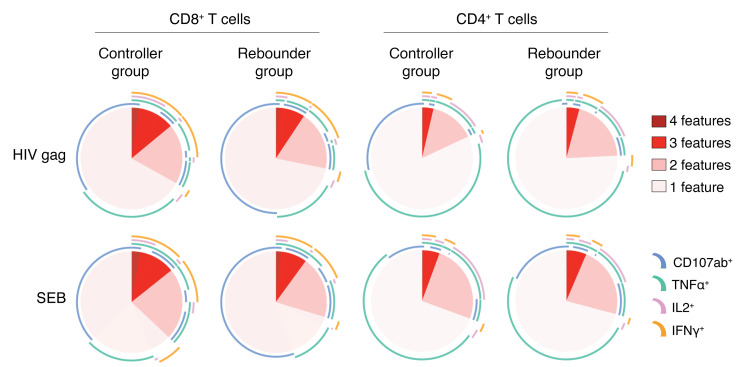
Combinatorial analysis of CD107a/b, TNF-α, IFN-γ, and IL-2 in the controller and rebounder group. SPICE diagrams reflecting proportions of SEB− or HIV-1 Gag-specific CD4^+^ and CD8^+^ T cell responses with indicated functional profile (determined by Boolean combination gating) in the controller and rebounder groups in a combinatorial analysis of data from PBMC samples collected at the beginning and at the end of Step 2. Pie chart colors represent number of effector molecules, whereas each effector function is represented by an arc.

To explore the potential effects of bnAbs on HIV-1–specific T cell responses in young children, we performed a phenotypic and functional analysis of CD4^+^ and CD8^+^ T cells in participants from the Tatelo cohort. Overall, there were no quantitative differences in global CD4^+^ or CD8^+^ T cells between the controller and rebounder groups (Supplemental Figure 6, A–C). Analysis of activation-induced surface markers (CD69, CD25, CD40L, OX40), degranulation markers (CD107a/b), or intracellular cytokines (IFN-γ, IL-2, TNF-α) following in vitro stimulation with overlapping clade C gag peptides demonstrated relatively few HIV-specific CD4^+^ and CD8^+^ T cell responses in both groups, with no significant differences between controllers and rebounders at the time of ART discontinuation or after reinitiation of ART (Figure 12, A and B). This was also true when ensemble cytokine secretion patterns or combinations of activation-induced surface marker expression profiles were considered (Figure 13). There was no evidence for an expansion or increase of HIV-1–specific CD4^+^ or CD8^+^ T cell responses during bnAb treatment. However, because our study was not designed to continue bnAb treatment for any children with viral rebound greater than 400 copies/mL, there was a limited opportunity to assess T cell responses with both bnAbs and detectable virus present. We also found no statistically significant association between HIV-1–specific CD4^+^ and CD8^+^ T cells and the corresponding frequencies of total, intact, or defective proviruses or with kinetics of viral rebound in the rebounder study group. This finding was limited by the fact that only 2 controllers initiated the study with detectable intact virus, and no controllers had detectable intact virus at the end of bnAb treatment. As shown in our previous work (45), HIV-1 antibody levels were generally low in the EIT cohort, with gp120-specific antibodies being almost entirely undetectable; this is likely due to the very early onset of ART in EIT study participants that intercepts complete seroconversion. Therefore, we did not perform additional studies to determine how viral rebound might be influenced by autologous neutralizing antibody responses.

## Discussion

The Tatelo study was the first clinical trial to evaluate the effects of dual bnAb therapy in a cohort of infants and young children with HIV-1. This study provided a unique opportunity to investigate antiviral immune responses and changes in the persistent viral reservoir during and after bnAb treatment in this specific population. We identified distinct subgroups of natural killer (NK) cells that correlated with viral rebound kinetics and observed that particular immunogenetic variations influencing NK cell education and function were linked to sustained viral control during bnAb-only therapy. In contrast, HIV-1–specific T cells showed no evidence of association with viral persistence or control following the cessation of standard antiretroviral therapy (ART) in these early ART-treated children with low viral reservoirs. Together, these findings suggest a role for NK cells in modulating viral rebound in young children undergoing bnAb treatment.

In our cohort of infants with pre-/perinatal HIV-1 acquisition, we found that higher proportions of KIR2DL1-expressing NK cells were associated with a shorter time to viral rebound during bnAb-only treatment, and these cells tended to be elevated in the rebounder group, particularly at the time of ART reinitiation. These results were complemented by a trend for higher frequencies of reservoir cells in carriers of HLA-C2 alleles, which encode for the predominant ligands for KIR2DL1, and by an enrichment tendency of rebounders with HLA-C2 allotypes, specifically those with high-level HLA-C2 surface expression. These data seem to correspond well with previous studies demonstrating an expansion of KIR2DL1^+^ NK cells in HLA-C2 carriers living with HIV-1 (46), consistent with an HLA-C genotype-dependent expansion of KIR2DL1^+^ NK cells in the context of HIV-1 infection. Collectively, these results suggest that, in infants and young children, KIR2DL1/HLA-C2 interactions may promote viral rebound, presumably due to KIR2DL1-mediated inhibition of NK cells that reduces their ability to target infected cells. In addition, KIR-dependent education of NK cells in infancy is immature and not fully adapted to host HLAs, resulting in a functional hyporesponsiveness and a more limited ability of KIR-educated NK cells to recognize and target virally infected cells through the classic “missing self” signal (47–49). However, a full understanding of how KIR2DL1-expressing NK cells can influence HIV-1 immune control, in the presence or absence of treatment with bnAbs, will likely depend on a direct assessment of HLA-C expression on viral reservoir cells. Recent technology advances in single-cell proteogenomic profiling now permit such experiments (50) and demonstrated an upregulation of HLA-C in cells harboring intact proviruses in a recent study of individuals who started ART in early infection (27), implying protection of viral reservoir cells from KIR2DL1-educated NK cells. However, such an assessment was not possible in our study due to the very low frequencies of infected cells. Nevertheless, it is conceivable that a notable proportion of viral reservoir cells express high levels of HLA-C, which may provide an efficient barrier against cytotoxic effects of KIR2DL1-expressing NK cells.

Interestingly, we observed that NKG2A-expressing NK cells showed an opposite pattern compared with KIR2DL1-expressing NK cells and were positively linked to time to viral rebound following standard ART discontinuation, and inversely associated with viral reservoir cell pool sizes in our cohort of infants with perinatal HIV acquisition. Of note, the latter observation is in line with previous findings in recent studies in adults (51) in which higher levels of NKG2A-expressing NK cells were also associated with lower levels of HIV-1 DNA, suggesting that NKG2A-expressing NK cells act as an important factor of antiviral immunity in children and adults. These results occurred despite NKG2A being formally classified as an inhibitory receptor that restricts NK cell killing after engaging its physiological ligand HLA-E on target cells (52). Nevertheless, previous studies have shown that NKG2A-restricted NK cells can exhibit enhanced cytotoxicity against HIV-1–infected cells, for reasons that are currently being explored. One study demonstrated that NKG2A fails to recognize an HIV-1 capsid peptide presented by HLA-E, and therefore does not effectively block cytotoxic activities against HLA-E–expressing HIV-infected cells (53). Moreover, a relatively low expression of HLA-E on infected target cells (at least 20-fold lower than HLA-C) (44) may be insufficient to meaningfully restrict cytotoxic activities of NKG2A-expressing NK cells, relative to interactions between HLA-C2 and KIR2DL1. Subsequent studies demonstrated that the HLA-B-21M polymorphism, which was present in the majority of controllers and associated with a trend for higher NKG2A-expressing NK cells and lower viral reservoir sizes in the Tatelo study, can stabilize HLA-E expression and may promote NKG2A/HLA-E–dependent education of CD56^dim^ NK cells, which might be associated with improved effector functions of this NK cell subset (42, 54). Of note, recent work also highlighted that, relative to alternative HLA-E stabilizing signal peptides, the signal peptides harboring the HLA-B-21M polymorphism can reduce binding affinities of the HLA-B-21M/HLA-E complex to NKG2A, markedly reducing the inhibitory effects of NKG2A towards HLA-E–expressing target cells and lowering the activation threshold for NK cells (43). Together, these results suggest a close interplay between NK cells and HIV-1–infected cells in young children and support the role of NK cells for HIV-1 immune control suggested by a number of recent studies (55–58). Future studies are warranted to better define how NK cells can specifically contribute to immune selection pressure against viral reservoir cells, in children and in adults.

In adults and in model systems involving nonhuman primates, immune effects of bnAbs were associated with an induction or expansion of HIV-1–specific T cell responses, presumably as a result of bnAb-mediated dendritic cell activation that then translates into enhanced immunostimulatory effects and improved HIV-specific T cell priming (59). However, in the Tatelo study, we found no evidence of such a vaccinal effect, and T cell responses were generally low in magnitude and unrelated to viral reservoir cell dynamics or viral rebound kinetics. We also found no signs of bnAb-induced activation in myeloid dendritic cells in Tatelo study participants. The absence of these responses may be linked to limited HIV exposure in early life due to very early ART initiation in the Tatelo study participants. However, it is also possible that these observations are related to age-specific differences in study cohorts, to a lower susceptibility of young children to bnAb-induced immune effects on T cells, or to differences in the immunogenicity of bnAbs in persons living with clade C versus clade B infection. We also acknowledge that our study cohort was strongly enriched for females, which may have influenced study outcomes; indeed, sex differences were shown to influence HIV-1 immune responses and viral reservoir dynamics in children (60), and in adults (61, 62). Ongoing and future studies of bnAbs in children will offer further opportunities to investigate effects of bnAbs on T cell responses. This includes the planned “Tatelo Plus” study, which will focus on administrating a combination of 3 different bnAbs to young children with HIV-1 from Botswana. These studies may also help determine whether the association of NK cell responses with viral rebound is reproducible in larger cohorts and identify biomarkers predictive of sustained viral control during bnAb therapy in infants and young children (63).

In addition to immune profiling experiments, we were also able to conduct assays to characterize the chromosomal locations of intact proviruses in some participants from whom sufficient cell samples were available. This approach, facilitated by technology developed by us and others (26, 64, 65), revealed that, in controller participants, intact proviruses were more frequently integrated into heterochromatic regions, particularly noncoding genomic regions, centromeric DNA, and ZNF genes, compared with defective proviruses. These findings recapitulate prior work in adults (31) and imply that host immune activity in infants and young children is sufficiently strong to mount selection pressure against proviruses that are integrated in genic locations and more likely to be transcriptionally active. To the extent that chromosomal integration site profiling was possible in our work, we observed that proviruses in heterochromatin locations appeared relatively early after treatment initiation, within 2–3 years of suppressive ART, in contrast to the approximately 15–20 years of ART required to observe similar patterns in adults (31). Together with the immune profiling data, these results support the hypothesis that HIV-1 reservoir cells in children are vulnerable to host immune activity (66). A deeper understanding of the host factors that can target and select HIV-1 reservoir cells in infants and young children may ultimately permit to strategically develop customized interventions to clear the majority of infected cells in clinical settings.

Studying the evolution of the HIV-1 reservoir in our participants posed significant technical challenges. Individuals recruited to this study acquired HIV prenatally and started ART near birth, leading to a very low persisting reservoir of genome-intact proviruses, consistent with prior results (20, 25, 67). The amount of PBMCs that can be safely collected from small children is low, which reduced our ability to adequately profile immune responses and viral reservoir cells. The Tatelo Study enrolled only 25 children in its bnAb-only phase, and interpretations were limited by the lack of a control group that did not receive bnAbs during ART interruption. There were several nonimmunologic factors associated with viral control during the intervention, including birth viral reservoir size, fewer viral blips or rebounds in early life, and susceptibility to 10-1074 (24). The present analysis suggests a possible role of NK cells in viral control based on differences between controllers and rebounders, but all associations remain difficult to disentangle. Finally, we emphasize that observations in the Tatelo cohort cannot easily be extrapolated to the adults setting, given that NK cell phenotypes mature and adjust with age (68); how NK cells influence viral reservoir sizes and viral rebound in adults, in the presence of absence of bnAb therapy, will require further investigation in future studies.

In summary, the development of optimized therapeutic approaches for children living with HIV remains an important unmet medical need (23, 69–72), and identifying strategies that may lead to posttreatment control are especially important. Children may be ideal candidates for novel posttreatment control strategies and can benefit throughout their entire lives once such strategies emerge. We believe that this study provides the first insights into NK cell features associated with viral reservoir dynamics and rebound kinetics during bnAb therapy in young children. Our findings advance the understanding of innate immunity in pediatric HIV infection and highlight a promising direction for future therapeutic strategies aimed at durable viral control after treatment interruption.

## Methods

### Sex as a biological variable.

Both male and female children were enrolled for the Tatelo study. The sex distribution within study participants is listed in Supplemental Table 1. While sex might impact viral control under bnAb therapy, sex was not considered as a variable in this study.

### Study design and approval.

PBMC samples were collected in Botswana from participants of the Tatelo Study (NCT03707977) who were enrolled into Step 2 of the Study. Details of the Tatelo Study Design were previously described (2). Study protocols were approved by the Botswana Ministry of Health’s Human Research Development Council, the Harvard T. H. Chan School of Public Health, and the Institutional Review Board of the Brigham and Women’s Hospital.

### Sample processing.

Blood samples from infants were collected using heel sticks or venipuncture. Blood samples were subjected to PBMC isolation using standard Ficoll-Paque density gradient centrifugation and cryopreserved according to standard protocols.

### Whole-genome amplification.

Extracted DNA was diluted to single HIV-1 genome levels so that 1 provirus was present in approximately 20%–30% of wells. Subsequently, DNA in each well was subjected to multiple displacement amplification (MDA) with phi29 polymerase (QIAGEN REPLI-g Single Cell Kit) per the manufacturer’s protocol. After this unbiased whole genome amplification, DNA from each well was split and separately subjected to near-full genome sequencing and integration site analysis. A second MDA amplification was performed to increase the DNA yield if necessary.

### Near-full-length genome sequencing of HIV.

Genomic DNA was diluted to single genome levels and subjected to HIV-1near-full genome amplification using a 1-amplicon, 2-amplicon, 3-amplicon or 5-amplicon approach (3) with primer sets adjusted to clade C sequences, as described previously (4). PCR products were visualized by agarose gel electrophoresis. Amplification products were subjected to Illumina MiSeq sequencing at the Massachusetts General Hospital (MGH) DNA Core facility. Resulting short reads were de novo assembled using Ultracycler v1.0 and aligned to HXB2 to identify large deleterious deletions (< 8000 bp of the amplicon aligned to HXB2), out-of-frame indels, premature/lethal stop codons, internal inversions, or 50-LTR defect (R15 bp insertions and/or deletions relative to HXB2), using an automated in-house pipeline written in Python scripting language (https://github.com/BWH-Lichterfeld-Lab/IntactnessPipeline; commit ID 17fde86). The presence or absence of APOBEC3G/3F–associated hypermutations was determined using the Los Alamos HIV Sequence Database Hypermut 2.0 program (5). Viral sequences that lacked all defects listed above were classified as ‘‘genome intact.” Multiple sequence alignments were performed using MAFFT. Phylogenetic distances between sequences were examined using Clustal X–generated neighbor-joining algorithms (6). Viral sequences were considered clonal if they had completely identical consensus sequences; single-nucleotide variations in primer binding sites were not considered for clonality analysis. When viral DNA sequences were undetectable, data were reported as LOD, calculated as 0.5 copies per maximum number of cells tested without target identification.

### Integration site analysis.

Integration sites were obtained employing integration site loop amplification (ISLA), using a protocol previously described (7); DNA produced by whole-genome amplification was used as a template. Resulting PCR products were subjected to next-generation sequencing using Illumina MiSeq. MiSeq paired-end FASTQ files were demultiplexed; small reads (142 bp) were then aligned simultaneously to the human reference genome GRCh38 and HIV-1 reference genome HXB2 using bwamem (8). Biocomputational identification of integration sites was performed according to previously described procedures (7). Briefly, chimeric reads containing both human and HIV-1 sequences were evaluated for mapping quality based on (a) HIV-1 coordinates mapping to the terminal nucleotides of the viral genome, (b) absolute counts of chimeric reads, (c) depth of sequencing coverage in the host genome adjacent to the viral integration site. The final list of integration sites and corresponding chromosomal annotations was obtained using Ensembl (v86, www.ensembl.org), the UCSC Genome Browser (www.genome.ucsc.edu), and GENCODE (v39, www.gencodegenes.org). Repetitive genomic sequences harboring HIV-1 integration sites were identified using RepeatMasker (www.repeatmasker.org).

### Flow cytometry.

PBMCs were thawed, stained with LIVE/DEAD Blue Viability Dye (Invitrogen) for 15 minutes, and subsequently preincubated for 10 minutes with of FcR blocking reagent (Miltenyi). Afterward, cells were incubated for 30 minutes with different combinations of appropriately titrated antibodies directed against HLA-DR (G46-6, BD Biosciences), CD3 (SK7, BD Biosciences), CD4 (SK3, BD Biosciences), CD8 (RPAT8, BD Biosciences), CD19 (SJ25C1, BD Biosciences), CD20 (2H7, BD Biosciences), CD203c (NP4D6, BD Biosciences), CD34 (563, BD Biosciences), CD158e1 (DX9, BD Biosciences), CD158f (UP-R1, Biolegend), TIGIT (REA1202, Miltenyi), NKp46 (9E2, Biolegend), CD96 (NK92.39, Biolegend), CD14 (M5E2, BD Biosciences), CD86 (2331, BD Biosciences), NKp30 (P30-15, Biolegend), CD123 (6H6, Biolegend), DNAM-1 (11A8, BD Biosciences), CD57 (NK-1, BD Biosciences), CD141 (1A4, BD Biosciences), NKG2A (131411, BD Biosciences), CD94 (HP-3D9, BD Biosciences), NKG2D (1D11, BD Biosciences), PD-1 (NAT105, BD Biosciences), CD11c (3.9, ThermoFisher), CD56 (B159, BD Biosciences), CD16 (3G8, BD Biosciences), CD158b (CH-L, BD Biosciences), CD158 (HP-MA4, Biolegend), CD1c (F10/21A3, BD Biosciences), NKG2C (134591, BD Biosciences), CD161 (HP-3G1, BD Biosciences). Subsequently, the cells were fixed in 2% paraformaldehyde in phosphate-buffered saline (PBS) and acquired on a Cytek Aurora spectral cytometer (Cytek) at the Ragon Institute Imaging Core Facility at MGH. To analyze HIV-1–specific T cell responses, PBMCs were rested in R10 medium for 4 hours at 37°C in 5% CO_2_ and then incubated with an HIV-1 consensus clade C Gag peptide pool (mix of 121 overlapping 15-mer peptides at 2 μg/mL for each peptide, NIH AIDS Reagent Program #12756) or SEB at 0.4 μg/mL (Sigma-Aldrich) in the presence of anti-CD28 and anti-CD49d at 1 μg/mL (BD Bioscience) and antibodies against CD107a and CD107b (clones H4A3 and H4B4, respectively, Biolegend). After 18 hours of incubation, brefeldin A at 5 μg/mL (BioLegend) and monensin at 1 μg/mL (BD Bioscience) were added, and cells were incubated for an additional 4 hours. An unstimulated negative control that lacked antigenic peptides but was otherwise treated identically was included for each sample. After stimulation, cells were washed and stained with LIVE/DEAD Blue Viability Dye (Invitrogen), followed by preincubation with 2 μl of FcR blocking reagent and surface staining for 20 minutes with antibodies directed against OX40 (ACT35, BD Biosciences), CD3 (UCHT1, BD Biosciences), CD8a (RTA-T8, Biolegend), CD40L (24-31, Biolegend), CD69 (FN50, BD Biosciences), CD25 (BC96, Biolegend), CD4 (RPA-T4, BD Biosciences). Subsequently, cells were washed, fixed, and permeabilized using the FoxP3 transcription factor buffer kit (eBioscience) for 30 minutes at 4°C. Intracellular cytokine staining was performed with antibodies directed against Perforin (B-D48, Biolegend), T-bet (REA102, Miltenyi), TOX (REA473, Miltenyi), Granzyme A (CB9, Biolegend), IFNγ (4S.B3, Biolegend), IL-2 (MQ1-17H12, Biolegend), Granzyme B (GB11, BD Bioscience), TNFα (Mab11, Biolegend) for 30 minutes at 4°C. The cells were fixed in 2% paraformaldehyde in PBS and acquired on a Cytek Aurora spectral cytometer (Cytek). Unstimulated controls were run for each sample and subtracted as background. Data were analyzed using FlowJo v.10.9.0 software (Tree Star LLC) and using the Simplified Presentation of Incredibly Complex Evaluations (SPICE) software (version 6.0) (9); gating strategies are summarized in Supplemental Figures 4 and 5.

To address unspecific staining of KIR2DL1 with the CD158 (HP-MA4, Biolegend) antibody that also binds to KIR2DS1, KIR2DS3, and KIR2DS5, we conducted additional experiments with limited samples that were available from the start of step 1, using specimens from study participants who were genotypically positive for KIR2DS1 and KIR2DS5. Using the KIR2DL1-specific antibody CD158a (HP-DM1, Biolegend) with the same staining protocol and flow cytometer, we did not determine significant differences in frequencies of KIR2DL1-expressing NK cells using either one of these 2 antibodies.

### Statistics.

Experimental variables between 2 groups of participants were analyzed using a 2-sided Mann-Whitney U test or a Wilcoxon matched-pair rank test, as appropriate. Differences were tested for statistical significance between 3 or more groups using the 2-sided Kruskal-Wallis nonparametric test with post-hoc Dunn’s multiple comparison tests or using false discovery rate calculations. Statistical associations were assessed using linear regression tests. All statistical analyses were performed using GraphPad Prism 10.2.2 and SPICE software.

### Study approval.

The study was approved by Institutional Review Boards in Botswana (Human Research Development Committee, HPDME 13/18/1 X1) and at the Harvard T. H. Chan School of Public Health (Harvard Longwood Campus Institutional Review Board, IRB18-0062). A parent or guardian provided written informed consent for all participants. The study was monitored by an independent Safety Monitoring Committee.

### Data availability.

All data associated with this study are present in the paper or the Supplemental Materials. Values for all data points in graphs are reported in the Supporting Data Values file. Deidentified or partially deidentified data, as appropriate, will be made available after the completion of the study to researchers with an approved protocol who complete a data use agreement. All inquiries should be sent to the corresponding author.

## Author contributions

Concept, design, and discussion: AN, XGY, RLS, DRK, and M Lichterfeld. Whole-genome amplification and HIV-1 sequencing: AN, SH, and RH. Integration site analysis: IR, LC, CN, and EP. Immune phenotyping and immunology assays: AN, CAH, M Lancien, TST. Bioinformatics analysis: AN and CG. Immunogenetic analysis: MC. Conduction of Tatelo clinical trial: KM, GA, SM, TM, CM, JM, OB, MPH, MSM, KMP, SL, and RLS. Data interpretation, analysis, and presentation: AN, CG, RLS, XGY, and M Lichterfeld. Supervision of immunological and virological experiments: XGY and M Lichterfeld. Manuscript writing, review, and editing: AN, RLS, DRK, XGY, and M Lichterfeld.

## Funding support

This work is the result of NIH funding, in whole or in part, and is subject to the NIH Public Access Policy. Through acceptance of this federal funding, the NIH has been given the right to make the work publicly available in PubMed Central.

- U. S. National Institutes of Health grant AI135940 (to RS, DK and M Lichterfeld).
- NIH grants AI176579, AI184094, AI155233, AI152979, AI155171.
- The Frederick National Laboratory for Cancer Research, under Contract No. 75N91019D00024.
- The Intramural Research Program of the NIH.
- Frederick National Lab, Center for Cancer Research.
- The NIH Fogarty International Center K43 TW012350 (SM).

## Supplementary Material

Supplemental data

Supplemental table 6

Supporting data values

## Version 1 02/16/2026

Electronic publication
