# Supplementary material for: Immune correlates of HIV-1 rebound during broadly neutralizing antibody treatment in young children
Source: J Clin Invest. 2026 Feb 16;136(4):e193912. doi: 10.1172/JCI193912 (PMC12904711; doi:10.1172/JCI193912)
Supplement: Supplemental data [file jci-136-193912-s281.pdf]

Supplementary Material

Table S1: Clinical and demographical data of Tatelo study participants

**Female sex, no. (%)**	19 (68)
**Age at ART start (days)**	3 (2-4)
**Age at bnAb start (years)**	3.7 (3.10 – 4.40)
**HIV-1 RNA at birth (copies/ml)**	3145 (310 – 25,507)
**CD4 T-cell count at start of bnAb-only treatment (cells/mm^3)**	1149 (922-1502)

Table S2: HLA class I types of the Tatelo study participants

Participant ID	Group	HLA-A	HLA-B	HLA-C
1	controller	02:02; 23:01	14:02; 15:10	03:04; 08:02
7	controller	23:01; 30:02	44:03; 45:01	02:10; 16:01
9	controller	43:01; 43:01	41:01; 44:03	02:10; 17:01
10	controller	30:02; 68:02	15:10; 57:03	03:04; 18:02
11	controller	23:01; 30:02	08:01; 58:02	07:01; x
16	controller	30:02; 30:02	08:01; 45:01	07:01; 16:01
17	controller	02:05; 68:02	14:02; 15:10	08:02; 08:04
18	controller	02:05; 43:01	39:10; 58:01	07:01; 15:05
22	controller	30:01; 68:02	42:01; 58:02	06:02; 17:01
29	controller	03:01; 23:01	15:03; 58:02	02:10; 06:02
36	controller	33:03; 36:01	42:01; 53:01	04:01; 17:01
2	rebounder	68:02; 74:01	15:03; 81:01	02:10; 18:01
13	rebounder	23:01; 74:01	15:10; 35:01	04:01; 16:01
15	rebounder	01:01; 02:05	58:01; 58:01	07:01; 07:01
19	rebounder	02:02; 30:04	53:01; 58:02	04:01; 06:02
20	rebounder	68:02; 68:02	15:03; 15:18	05:01; 07:01
24	rebounder	02:02; 23:01	08:01; 14:02	07:02; 08:02
26	rebounder	33:03; 74:01	35:01; 53:01	04:01; 04:01
30	rebounder	23:01; 30:02	44:03; 57:03	02:10; 18:02
31	rebounder	02:05; 68:02	41:02; 44:03	04:01; 17:01
32	rebounder	01:01; 23:01	08:01; 81:01	07:01; 18:01
33	rebounder	03:01; 30:01	15:03; 58:02	02:10; 06:02
35	rebounder	23:01; 32:106	41:01; 45:01	06:02; 17:01
40	rebounder	29:02; 29:02	15:03; 58:02	02:10; 06:02
201	rebounder	30:01; 32:106	15:220; 42:02	04:01; 17:01

Table S3: Study participants carrying KIR genes

	Controller (%)	Rebounder (%)
**KIR2DL1**	100	93
**KIR2DL2**	73	73
**KIR2DL3**	82	64
**KIR3DL1**	100	93
**KIR2DS1**	9	29

Table S4: Demographic data of reference cohorts.

Cohort	Participant numbers	Male %	Age	CD4 T cell count/µl	Recorded duration of undetectable viremia (years)	Time since diagnosis (years)
EC	72	81.9	57 (31-75)	882 (450-2282)	8.8 (1-24.2)	17.7 (1.3-34.4)
ART	43	69.8	55 (34-73)	727 (316-1657)	8 (1-19)	18 (1-35)
RIVER	5	100	44 (28-52)	724 (579-1051)		

Table S5: Reservoir data from the Tatelo cohort

Group	Timepoint	Analyzed PBMCs	Intact Proviruses	Defective Proviruses
controller	w0	1.11E+07	48	71
controller	w84	2.19E+07	8	76
controller	bnAb start	3.93E+07	10	151
controller	ART re-start	2.86E+07	0	80
controller	6-12 months post ART re-start	2.79E+07	1	8
controller	1-3 years post ART re-start	5.15E+07	11	645
rebounder	w0	6.34E+06	87	107
rebounder	w84	1.97E+07	13	53
rebounder	bnAb start	5.16E+07	5	125
rebounder	ART re-start	2.55E+07	16	77
rebounder	6-12 months post ART re-start	3.07E+07	17	89
rebounder	1-3 years post ART re-start	4.02E+07	14	117

Table S6: Integration Sites of Tatelo study participants
Separate Excel file.

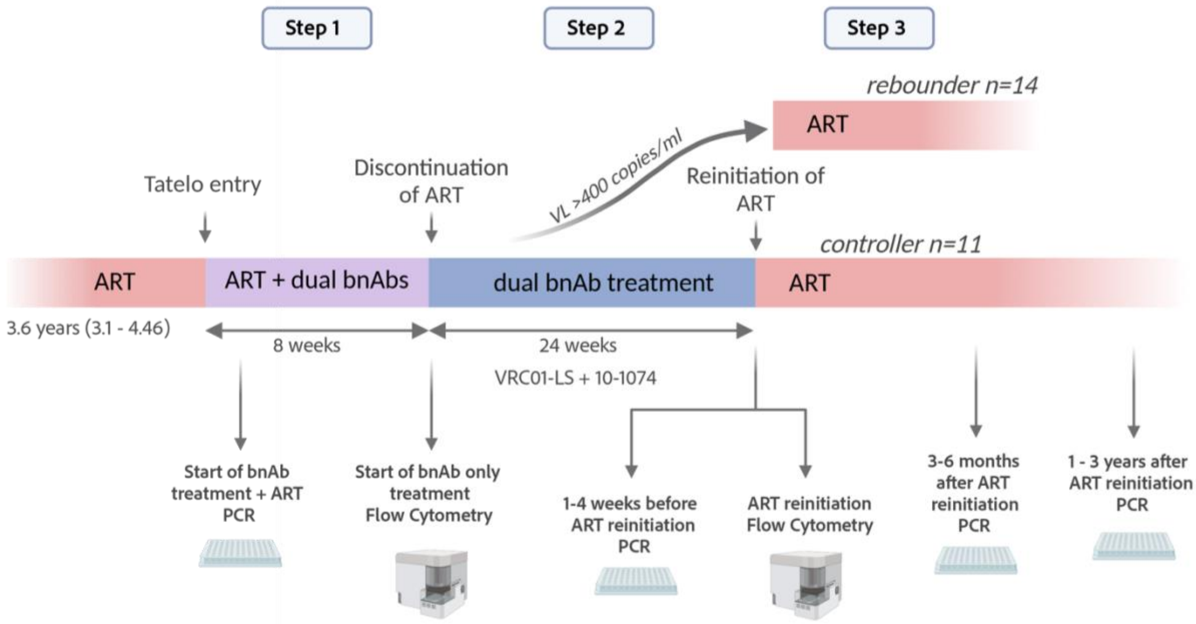

Figure S1: Schematic representation of the design of the Tatelo study.

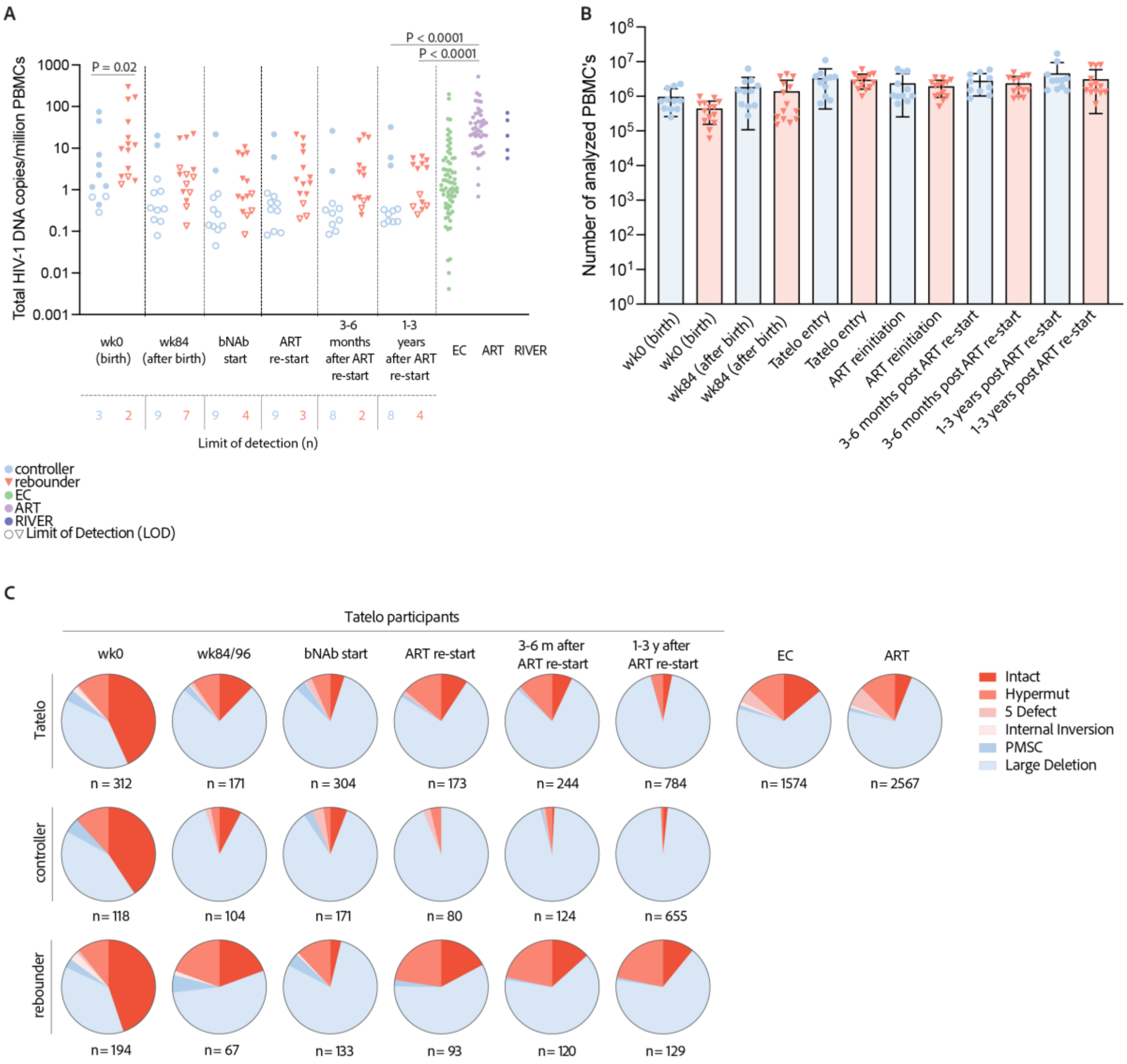

Figure S2. Viral reservoir diversity in early-treated children with dual bnAb treatment. (A) Frequency of total proviruses in controllers and rebounders from the Tatelo study, measured at indicated timepoints (wk0 = birth, wk84 = week 84 after birth). Data from elite controllers (EC), long-term ART-treated adults who initiated treatment during chronic infection, and adults who started ART during acute infection in the RIVER cohort (49) are presented for comparative purposes. Limit of defection (LOD) was calculated as 0.5 copies per maximum number of cells tested without target identification. Data was obtained by FLIP and MIP-seq. Two-tailed Mann-Whitney-U test was used. (B) Bar chart displaying the number of PBMC (analyzed by FLIP- or MIP-seq) per study participant in each group at indicated timepoints. (C) Longitudinal analysis of viral reservoir composition in Tatelo participants compared to Elite Controller and long-term ART-treated adult cohorts, as determined by single-genome near–full-length,

next-generation sequencing. Longitudinal data from timepoints at birth until 1-3 years after ART reinitiation are shown. Each pie chart reflects the relative contribution of HIV-1 amplification products with defined defects or genome-intact sequences. Total number of individual sequences included in each diagram is listed on the bottom of each pie chart.

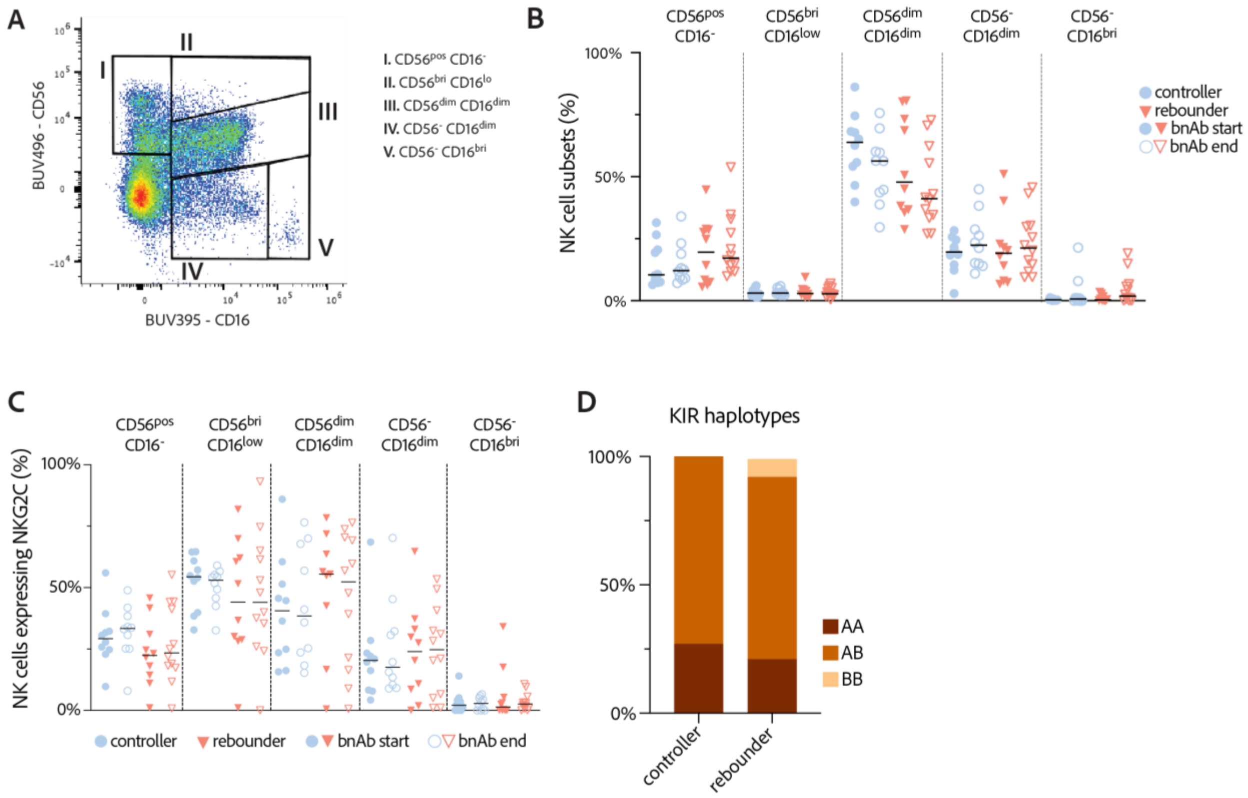

Figure S3: NK cell characterization in the controller and rebounder group. (A) Representative flow cytometry pseudocolor plot highlighting subclassification of NK cell subsets stratified according to CD16 and CD56 expression. (B) Proportions of NK cells with indicated CD16 and CD56 surface expression patterns in controller and rebounder groups. (C) Relative expression of NKG2C in indicated NK cell subsets in the controller and rebounder group at bnAb start and bnAb end. (D) Distribution of KIR haplotypes in the controller and rebounder group based on KIR genotypes in Table S3. (B/D) Medians are indicated by horizontal lines.

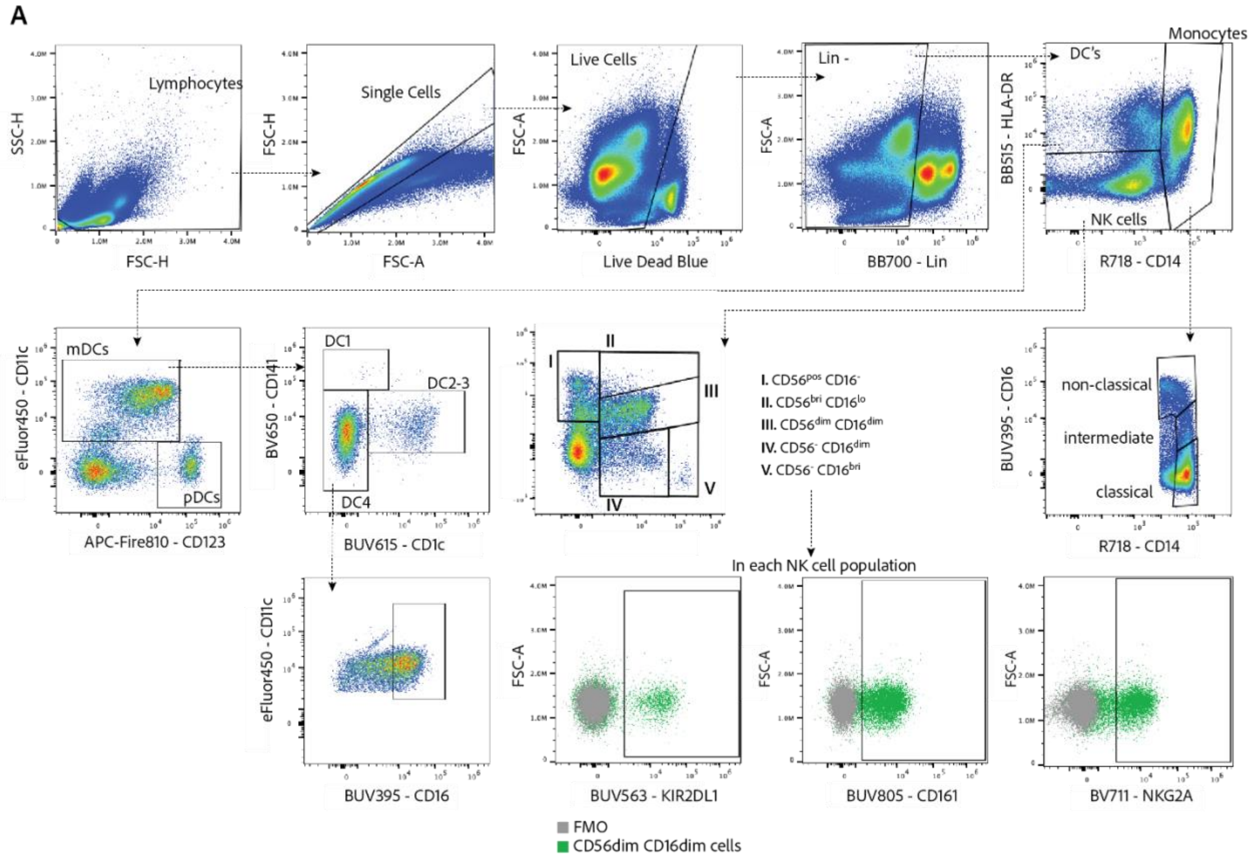

Figure S4. Gating Strategy for innate immune cell profiling. Sequential gating steps for analysis of NK cells, monocytes and dendritic cells are indicated.

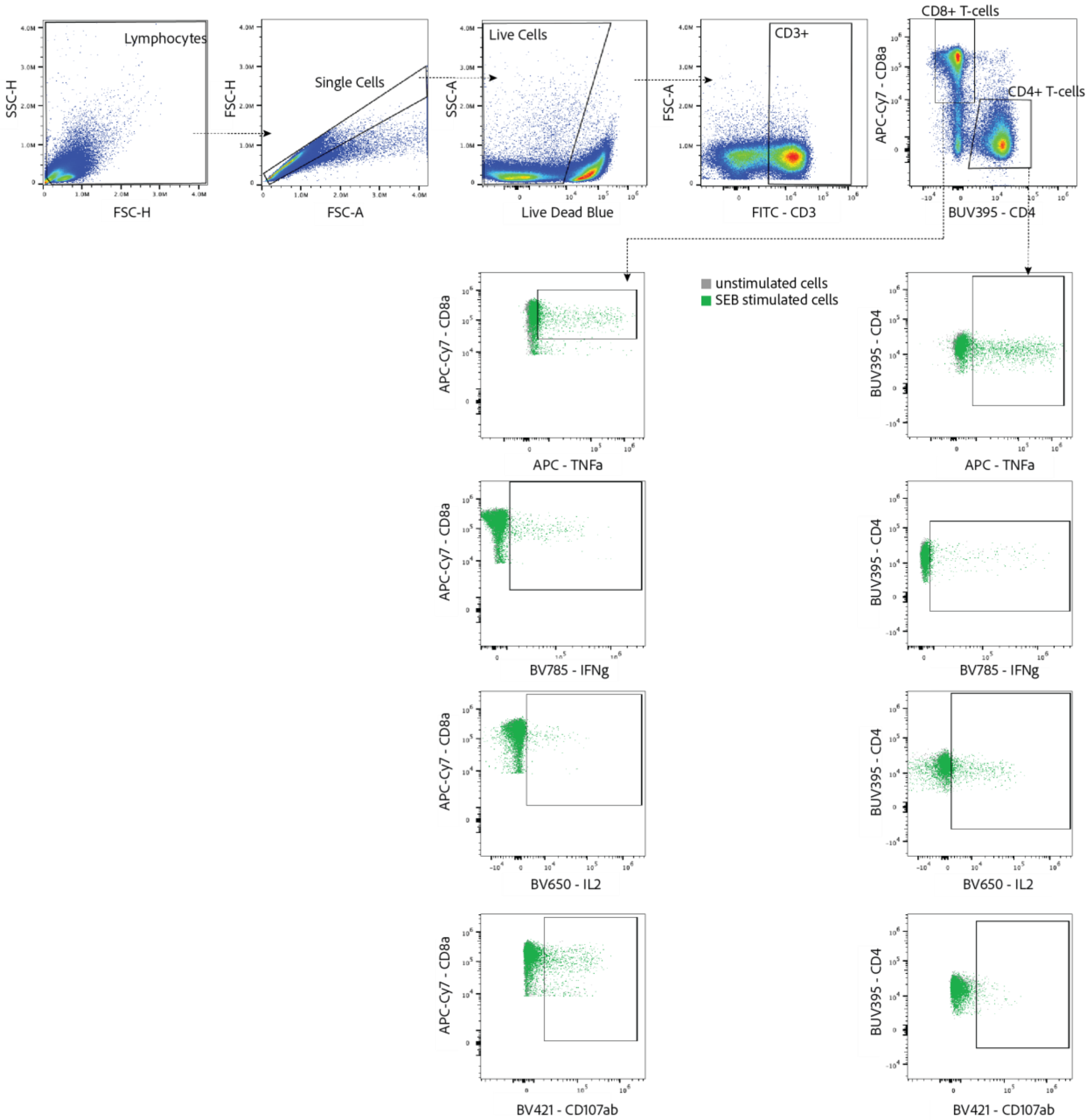

Figure S5. Gating Strategy for T-cell immune profiling. Sequential gating steps for analysis of antigen-specific CD4^{+} and CD8^{+} T-cells are shown.

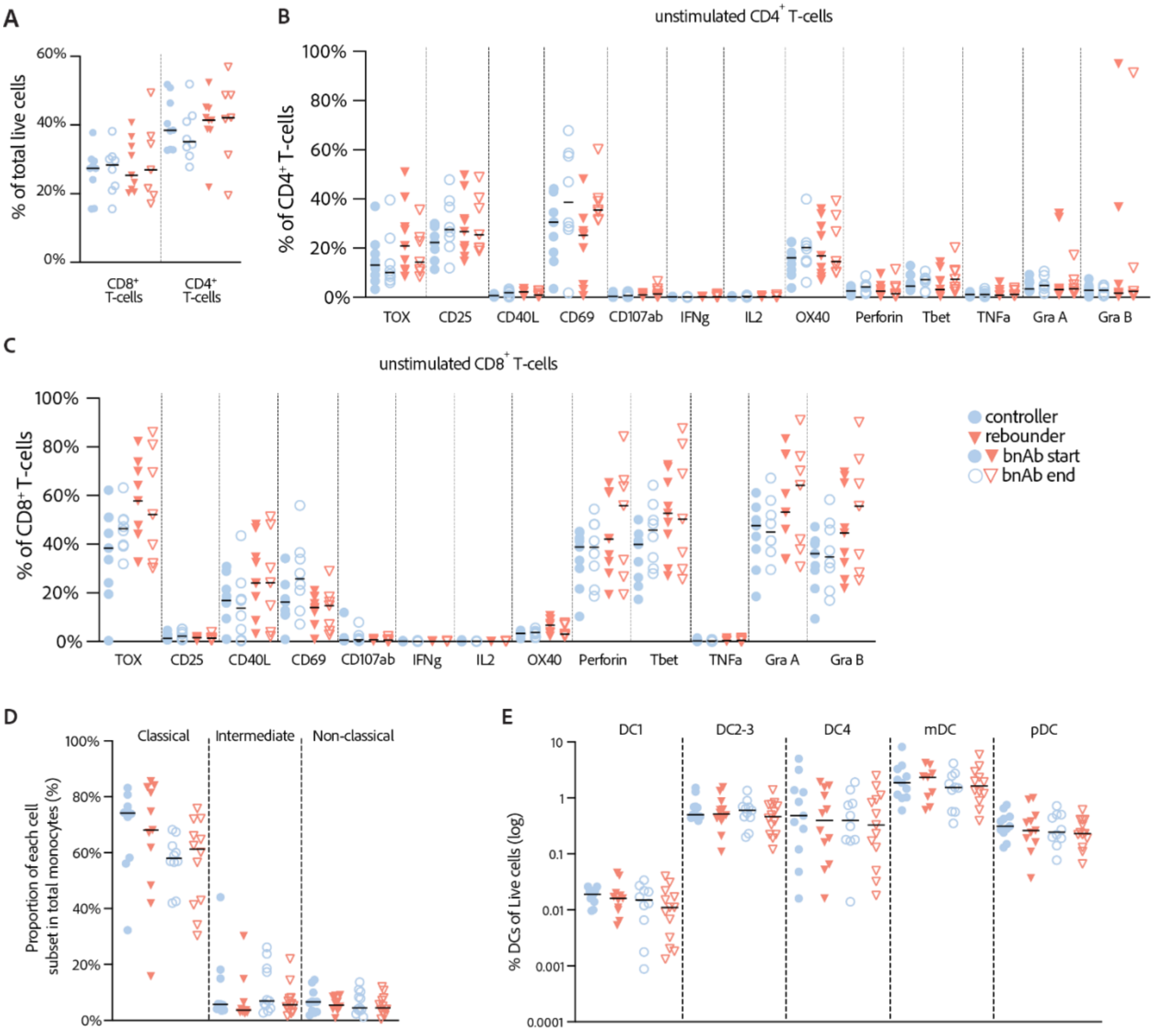

Figure S6: Proportions of unstimulated T-cells and myeloid cell populations in Tatelo study participants. (A) Proportions of total live CD4+ and CD8+ T cells in PBMC after HIV gag stimulation in the controller and rebounder group. (B-C) Proportions of CD4+ and CD8+ T cells expressing indicated activation-induced surface markers and intracellular cytokines in unstimulated PBMCs. (D) Proportions of monocyte subsets (defined by expression of CD14/CD16) at the beginning and at the end of bnAb-only treatment in both groups. (E) Proportions of DC subsets at the beginning and at the end of bnAb-only treatment in both groups. (A-D) Medians are indicated by horizontal lines.
